# Time to COVID-19 Vaccination by Language and Country of Origin

**DOI:** 10.1001/jamanetworkopen.2024.37388

**Published:** 2024-10-03

**Authors:** Margaret B. Nolan, Ella Chrenka, Malini B. DeSilva

**Affiliations:** 1 HealthPartners Institute for Medical Education and Research, Bloomington, Minnesota

## Abstract

**Importance:**

Disparities in COVID-19 vaccination rates by race and ethnicity are well documented. Less is known about primary language and COVID-19 vaccine uptake.

**Objective:**

To describe the time to COVID-19 primary series vaccination and booster doses by primary language and country of origin.

**Design, Setting, and Participants:**

This retrospective cohort study included patients aged 6 months or older with at least 1 health encounter from July 1, 2019, to June 30, 2023, at a single health care system serving patients across Minnesota and western Wisconsin.

**Exposure:**

Primary language and country of origin documented in the electronic health record.

**Main Outcomes and Measures:**

Three COVID-19 vaccine coverage outcomes were evaluated: (1) primary series (1 Ad26.COV.S vaccine or 2 mRNA COVID-19 vaccines), (2) first-generation booster (primary series Ad26.COV.S vaccine plus 1 Ad26.COV.S or mRNA COVID-19 vaccine at least 2 months after the second dose or primary series mRNA vaccine plus 1 mRNA vaccine at least 5 months after the second dose), and (3) bivalent booster. Vaccine coverage was described by patient characteristics. Associations of primary language, race and ethnicity, and other patient characteristics with COVID-19 vaccine uptake were evaluated using time-to-event analysis in multivariable Cox proportional hazards regression models, and adjusted hazard ratios (AHRs) with 95% CIs were reported.

**Results:**

There were 1 001 235 patients included (53.7% female). Most patients reported English as a primary language (94.1%) and were born in the US (91.8%). Primary series coverage was 63.7%; first-generation booster coverage, 64.4%; and bivalent booster coverage, 39.5%. Coverage for all outcomes was lower among those with a non-English primary language compared with English as the primary language (56.9% vs 64.1% for primary series; 47.5% vs 65.3% for first-generation booster; 26.2% vs 40.3% for bivalent booster). Those with a non-English primary language had lower COVID-19 vaccine uptake for the primary series (AHR, 0.85; 95% CI, 0.84-0.86), first-generation booster (AHR, 0.74; 95% CI, 0.73-0.75), and bivalent booster (AHR, 0.65; 95% CI, 0.64-0.67) compared with patients with English as their primary language. Non–US-born patients had higher primary series uptake compared with US-born patients (AHR, 1.19; 95% CI, 1.18-1.20) but similar first-generation booster (AHR, 1.01; 95% CI, 0.99-1.02) and bivalent booster (AHR, 1.00; 95% CI, 0.98-1.02) uptake.

**Conclusions and Relevance:**

In this retrospective cohort study, patients with a non-English primary language had both lower coverage and delays in receiving COVID-19 vaccines compared with those with English as their primary language. Reporting on language may identify health disparities that can be addressed with language-specific interventions.

## Key Points

**Question:**

Are there differences in COVID-19 vaccine uptake by primary language and country of origin?

**Findings:**

In this cohort study of 1 001 235 patients, those with a non-English primary language had a lower likelihood of primary series, first-generation booster, and bivalent booster completion compared with patients who had English as their primary language.

**Meaning:**

Reporting vaccination coverage and uptake by primary language may identify health disparities that can be addressed with language-specific interventions.

## Teaser

This cohort study evaluates whether time to COVID-19 vaccine uptake differs by primary language and country of origin among patients in a health care system serving Minnesota and western Wisconsin.

## Introduction

Disparities in COVID-19 vaccination rates by race and ethnicity are well documented.^1,2^ Reporting by broad race and ethnicity categories, however, fails to reflect the diversity of many individuals residing in the US.^3,4^ Recent research by Quadri et al^5^ and others^6,7^ found that language preference other than English was associated with delayed receipt of the first dose of COVID-19 vaccine and higher COVID-19 hospitalization rates compared with having English language preference. The current study built on the findings of studies like those of Quadri et al^5^ by adding time to mono- and bivalent booster doses, including in pediatric populations, and reporting time to vaccination by preferred language group. Qualitative research among patients with language preference other than English related to COVID-19 vaccines suggested that reliance on family members with English fluency was critical to receiving a timely first dose, and concerns about needing to show identification and insurance cards created initial barriers, particularly for refugee and immigrant patients.^8,9^ More time spent unvaccinated or undervaccinated means longer duration of risk for poor outcomes from COVID-19, and delays in vaccination may be partly responsible for some of the disparities noted in morbidity and mortality among racial and ethnic groups in the US.^4,5^

The aim of this study was to evaluate both COVID-19 vaccine coverage and uptake (ie, time to COVID-19 vaccination) by patient demographics, including primary language, country of origin, and need for an interpreter, to identify groups with lower coverage and delayed COVID-19 vaccination. Better understanding of the timing of vaccine uptake by these characteristics may help identify areas for future public health investment, education, and outreach. Differences in the rate of vaccine uptake over time can alert clinicians and public health workers to vaccine hesitancy or accessibility issues within a community as well as identify the need for specific education and outreach efforts.

## Methods

This retrospective cohort study evaluated COVID-19 vaccine administration between December 14, 2020, and June 30, 2023. It included patients receiving care at HealthPartners, a large health system servicing patients in Minnesota and western Wisconsin. The study received a determination of non–human participant research from the institutional review board at the HealthPartners Institute. The Strengthening the Reporting of Observational Studies in Epidemiology (STROBE) reporting guideline was followed.

The study population included patients aged 6 months or older as of June 30, 2022, who had been seen for a primary care visit at least once from July 1, 2019, through June 30, 2023. Three COVID-19 vaccine coverage outcomes were evaluated, defined as follows: (1) primary series (1 Ad26.COV.S) vaccine or 2 mRNA COVID-19 vaccines), (2) first-generation booster (primary series Ad26.COV.S vaccine plus 1 Ad26.COV.S or mRNA COVID-19 vaccine at least 2 months after or primary series mRNA vaccine plus 1 mRNA vaccine at least 5 months after the second dose), and (3) bivalent booster.

Patient data were extracted from the electronic health record (EHR). Self-reported variables include patient sex, race and ethnicity, primary language, and need for an interpreter. Race and ethnicity was grouped by non-Hispanic American Indian or Alaska Native, non-Hispanic Asian, non-Hispanic Black or African American, Hispanic or Latino, non-Hispanic Native Hawaiian or Pacific Islander, non-Hispanic White, and multiracial, which also included all other racial categories (unknown race and ethnicity). Race and ethnicity were included to show that race and ethnicity, although related, are not necessarily a marker of language or country of origin. Primary language was broadly defined as English or non-English. For 1 analysis, we also disaggregated primary languages into the 5 most common primary languages individually and grouped other languages into categories based on geographic areas. Need for an interpreter was defined by whether an interpreter was requested or not at the most recent primary care visit. Additional variables included age as of June 30, 2022, divided into groups (ie, 6 months to 4 years and 5-11, 12-16, 17-29, 30-49, 50-64, and ≥65 years), most recent insurance provider (private, public, other, or unknown), and presence of 1 or more immunocompromising conditions within the previous year, identified using previously developed definitions based on *International Statistical Classification of Diseases, Tenth Revision, Clinical Modification* codes.^10^ COVID-19 vaccine product and administration dates were extracted from the EHR and Minnesota’s state immunization information system.

### Statistical Analysis

The 3 COVID-19 vaccine coverage outcomes were calculated by patient characteristics. All patients were included in the primary series coverage evaluation. First-generation and bivalent booster coverage analyses were limited to patients with a completed primary series by December 31, 2022, to allow time for booster receipt, as booster doses were recommended to be administered 2 or more months after completing the primary series.^11^ Patients younger than 5 years were removed from the analysis of first-generation booster rates due to the timing of vaccine availability with less than 3 months between approval of the primary series (June 18, 2022) and bivalent booster approval (September 1, 2022) for this age group.

Survival analysis was used to model vaccine coverage outcomes over the study period, with records censored at the end of the observation period (ie, June 30, 2023). For the primary series, time to event was measured as days between December 14, 2020 (date when COVID-19 vaccines were first available), and the date of primary series completion. For the first-generation booster analysis, time to event was defined as the number of days between primary series completion and receipt of the first-generation booster dose agnostic to date. Bivalent booster time to event was measured as the number of days between September 1, 2022 (date when the Advisory Committee for Immunization Practices voted to recommend a single dose of bivalent COVID-19 mRNA vaccine), and receipt of bivalent booster.^12^

To account for differences in dates of vaccine eligibility based on patient age, age-adjusted cumulative vaccine uptake was visualized using inverse Kaplan-Meier curves by primary language (English, non-English, and the 5 most common primary languages individually) and country of origin (US vs non-US). To evaluate the association of primary language, country of origin, age, sex, race and ethnicity, insurance, and presence of immunocompromising conditions with the outcomes, 3 multivariable Cox proportional hazards regression models (1 for each vaccine coverage outcome) were used to calculate adjusted hazard ratios (AHRs) with 95% CIs. Need for an interpreter was excluded as a regressor from the models to avoid collinearity with primary language. Patients with missing language or birth country data were grouped with the reference level for the main analysis. A secondary analysis included models of vaccine uptake with disaggregated primary language. Sensitivity analyses included (1) models excluding those with missing race and country of origin data to evaluate the decision to include these in the reference group and (2) analysis of the bivalent booster outcome including all patients regardless of primary series completion to determine whether exclusion of those without a primary series completion influenced the primary findings. All analyses were conducted in R, version 4.2 (R Project for Statistical Computing).^13^

## Results

There were 1 001 235 patients included; 53.7% were female, and 46.2% were male. A total of 0.3% were American Indian or Alaska Native; 5.5%, Asian; 10.3%, Black or African American; 5.0%, Hispanic or Latino; 0.1% Native Hawaiian or Pacific Islander; 58.2%, White; and 14.7%, multiracial (Table 1). Most patients reported English as their primary language (94.1%) and were born in the US (91.8%). A total of 4.9% were younger than 5 years. Most patients (95.7%) had no immunocompromising conditions. Missing records for language, country of origin, and need for an interpreter were present for 1.5%, 26.6%, and 3.5%, respectively.

**Table 1.** Demographic Characteristics and COVID-19 Vaccine Coverage for Patients Aged 6 Months or Older From July 1, 2019, to June 30, 2023

Variable	Patients, No. (%)				
	Total	Primary series coverage^a^	Booster eligible	First-generation booster coverage^b^	Bivalent booster coverage^b^
Overall	1 001 235 (100)	637 397 (63.7)	635 539 (100)	408 978 (64.4)	251 191 (39.5)
Language					
English^c^	942 383 (94.1)	603 894 (64.1)	602 358 (94.8)	393 210 (65.3)	242 536 (40.3)
Non-English	58 852 (5.9)	33 503 (56.9)	33 181 (5.2)	15 768 (47.5)	8655 (26.1)
Country of origin					
US^d^	919 104 (91.8)	577 606 (62.8)	575 939 (90.6)	373 831 (64.9)	231 558 (40.2)
Non-US	82 131 (8.2)	59 791 (72.8)	59 600 (9.4)	35 147 (59.0)	19 633 (32.9)
Need for an interpreter					
No^e^	955 761 (95.5)	611 277 (64)	609 686 (95.9)	396 473 (65)	244 264 (40.1)
Yes	45 474 (4.5)	26 120 (57.4)	25 853 (4.1)	12 505 (48.4)	6927 (26.8)
Race and ethnicity					
Non-Hispanic American Indian or Alaska Native	2739 (0.3)	1635 (59.7)	1628 (0.3)	988 (60.7)	616 (37.8)
Non-Hispanic Asian	54 688 (5.5)	41 964 (76.7)	41 721 (6.6)	27 011 (64.7)	15 654 (37.5)
Non-Hispanic Black or African American	103 518 (10.3)	51 504 (49.8)	51 066 (8)	20 949 (41)	12 162 (23.8)
Hispanic or Latino	50 164 (5.0)	28 911 (57.6)	28 756 (4.5)	14 547 (50.6)	8251 (28.7)
Non-Hispanic Native Hawaiian or Pacific Islander	883 (0.1)	561 (63.5)	559 (0.1)	346 (61.9)	212 (37.9)
Non-Hispanic White	582 994 (58.2)	400 384 (68.7)	399 870 (62.9)	280 425 (70.1)	177 577 (44.4)
Multiracial	146 778 (14.7)	83 456 (56.9)	83 127 (13.1)	49 016 (59)	28 111 (33.8)
Unknown	59 471 (5.9)	28 982 (48.7)	28 812 (4.5)	15 696 (54.5)	8608 (29.9)
Sex					
Female	537 712 (53.7)	352 105 (65.5)	351 103 (55.2)	231 379 (65.9)	142 768 (40.7)
Male	463 009 (46.2)	285 085 (61.6)	284 229 (44.7)	177 471 (62.4)	108 340 (38.1)
Unknown or other	514 (0.1)	207 (40.3)	207 (<0.1)	128 (61.8)	83 (40.1)
Age, y^f^					
0.5-4^g^	49 068 (4.9)	7646 (15.6)	6648 (1)	16 (0.2)	2495 (37.5)
5-11	76 414 (7.6)	29 067 (38.0)	28 749 (4.5)	6650 (23.1)	9511 (33.1)
12-16	59 540 (5.9)	33 098 (55.6)	33 008 (5.2)	13 679 (41.4)	9973 (30.2)
17-29	182 963 (18.3)	109 297 (59.7)	109 065 (17.2)	56 722 (52)	25 789 (23.6)
30-49	289 350 (28.9)	194 758 (67.3)	194 586 (30.6)	123 153 (63.3)	64 570 (33.2)
50-64	186 841 (18.7)	141 039 (75.5)	141 004 (22.2)	103 989 (73.7)	62 265 (44.2)
≥65	157 059 (15.7)	122 492 (78)	122 479 (19.3)	104 769 (85.5)	76 588 (62.5)
Immunocompromising condition^h^					
No	958 614 (95.7)	600 745 (62.7)	598 909 (94.2)	379 245 (63.3)	229 175 (38.3)
Yes	42 621 (4.3)	36 652 (86)	36 630 (5.8)	29 733 (81.2)	22 016 (60.1)
Insurance					
None or unknown	51 835 (5.2)	17 551 (33.9)	17 501 (2.8)	7091 (40.5)	2611 (14.9)
Private	571 314 (57.1)	391 016 (68.4)	390 233 (61.4)	249 720 (64)	141 761 (36.3)
Public	378 086 (37.8)	228 830 (60.5)	227 805 (35.8)	152 167 (66.8)	106 819 (46.9)

^a^ Percentages were calculated with the row total as the denominator.

^b^ Percentages were calculated with the number of booster-eligible patients in each row as the denominator.

^c^ Includes those with unknown language (15 445 [1.5%]).

^d^ Includes those with unknown country of origin (266 157 [26.6%]).

^e^ Includes those with no recorded need for an interpreter (35 487 [3.5%]).

^f^ Age calculated as of June 30, 2022.

^g^ Patients of 4 or less removed from subsequent first-generation booster analysis.

^h^ Defined as least 1 diagnosis of solid malignancy, hematologic malignancy, rheumatologic or inflammatory disorder, or organ or stem cell transplant; other intrinsic immune condition; or immunodeficiency between January 1, 2019, and June 30, 2023.^8^

The overall primary series coverage was 63.7%, first-generation booster coverage was 64.4%, and bivalent booster coverage was 39.5%. Coverage was lower for all outcomes among those with a non-English primary language compared with those with English as their primary language (56.9% vs 64.1% for primary series; 47.5% vs 65.3% for first-generation booster; 26.2% vs 40.3% for bivalent booster) and those requesting an interpreter compared with those without need for an interpreter. Primary series completion was higher among those born outside the US compared with US-born persons but lower for booster coverage. Coverage was lowest among Black individuals compared with those in the other racial and ethnic groups. COVID-19 vaccine coverage increased with increasing age for the primary series and first-generation booster and was lowest in the 17- to 29-year age group for bivalent booster coverage (Table 1).

The median time to primary series completion was 190 days (range, 14-899 days), with 50.0% of those who completed the series receiving the full series by June 22, 2021. The median time from primary series completion to first-generation booster was 261 days (range, 59-763 days). Of those who received a bivalent booster by the end of the study period, the median time to uptake was 57 days (range, 0-302 days).

**Figure 1.**
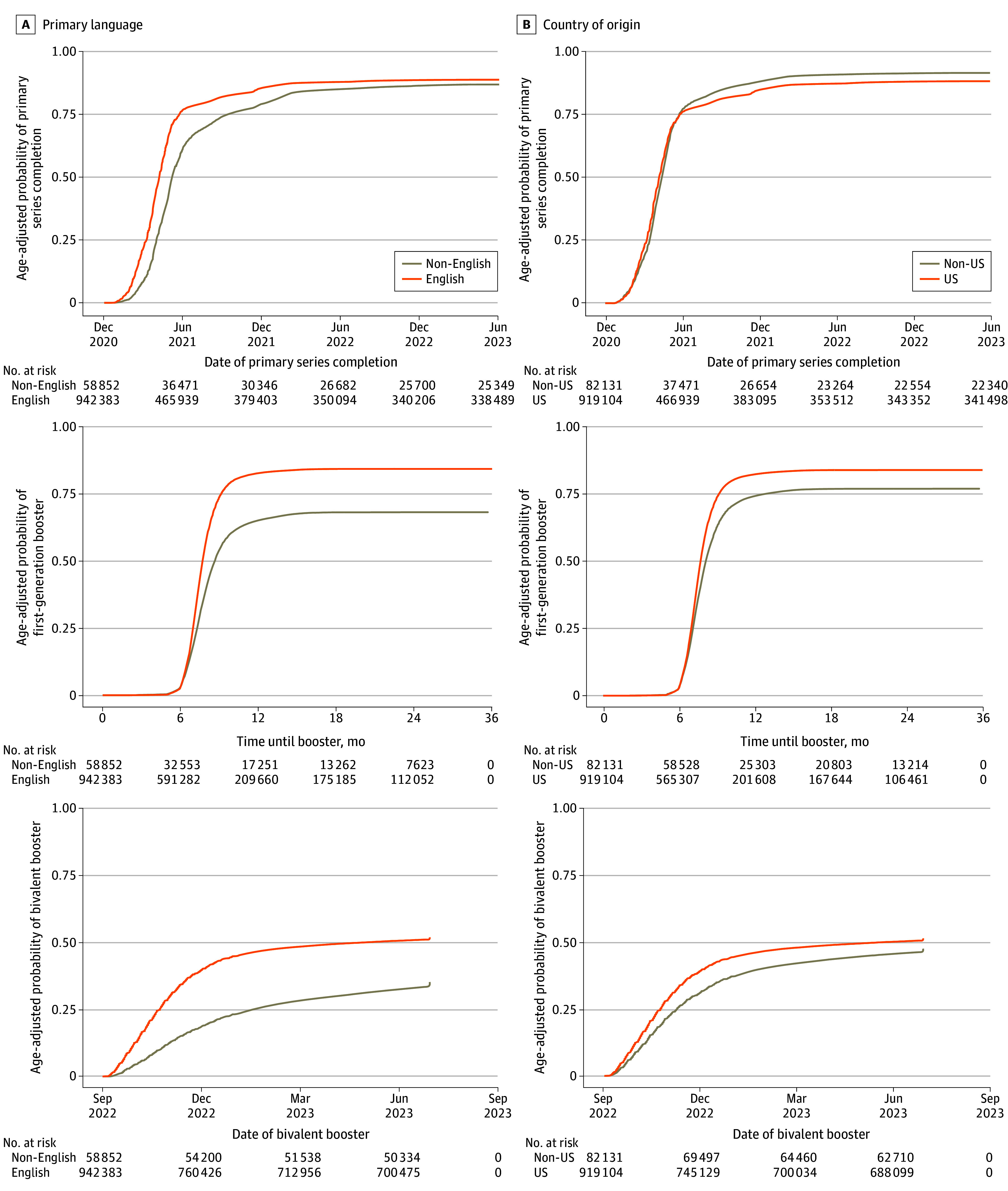
Kaplan-Meier Curves Comparing Age-Adjusted Uptake of COVID-19 Vaccines by Language and Country of Origin

**Figure 2.**
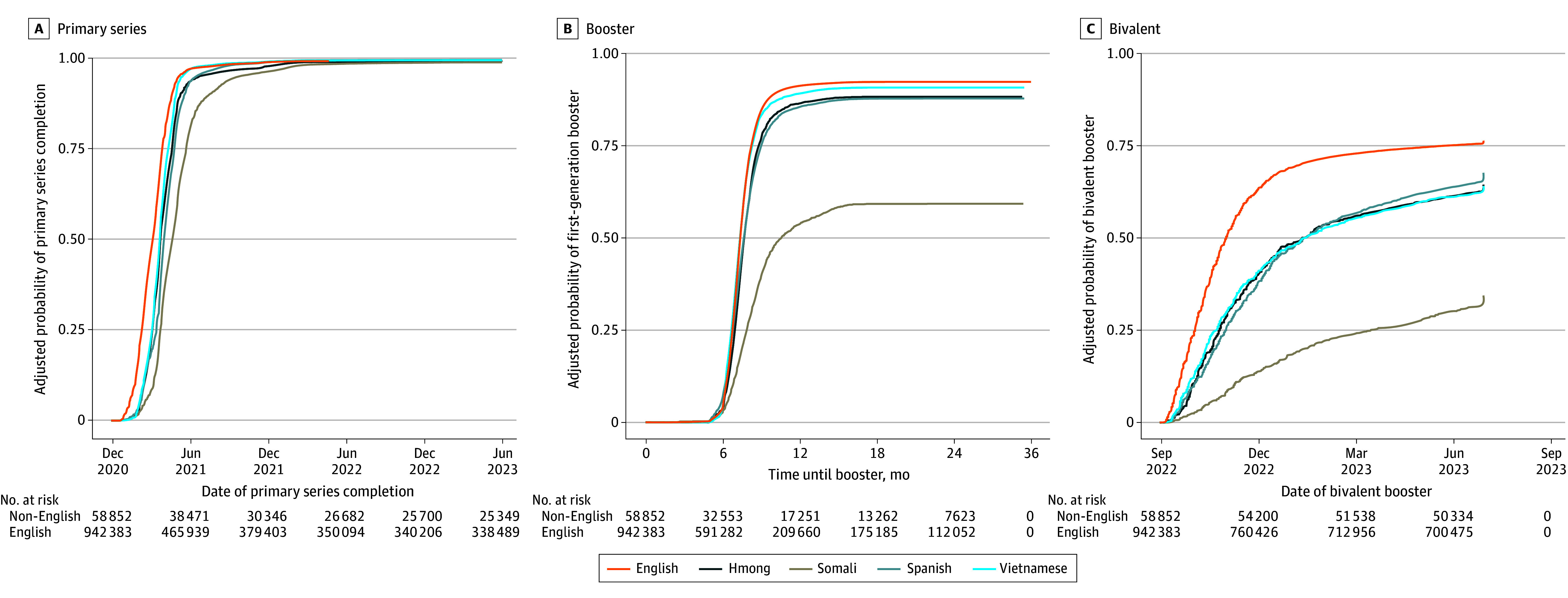
Kaplan-Meier Curves Comparing Age-Adjusted Uptake of COVID-19 Vaccines by the 5 Most Commonly Reported Languages Adjusted for patient sex, age, race and ethnicity, country of origin (US vs non-US), immunocompromised status, and insurance type.

Kaplan-Meier curves display the changes in cumulative vaccination coverage over time with adjustment for age and stratified by primary language and country of origin (Figure 1 and Figure 2). Patients with a non-English primary language had delayed rates of primary series and booster vaccination compared with those with English as their primary language. Non–US-born patients had higher primary series coverage than US-born patients but lower first-generation and bivalent booster coverage.

Patients with a non-English primary language had lower uptake of the primary series (AHR, 0.86; 95% CI, 0.85-0.87), first-generation booster (AHR, 0.74, 95% CI, 0.72-0.75), and bivalent booster (AHR, 0.65; 95% CI, 0.64-0.67) compared with those with English as their primary language (Table 2). Non–US-born patients had higher primary series uptake compared with US-born patients (AHR, 1.21; 95% CI, 1.20-1.22) but similar uptake of the first-generation booster (AHR, 0.99; 95% CI, 0.98-1.01) and bivalent booster (AHR, 0.99; 95% CI, 0.98-1.01). Patients from racial and ethnic minority groups excluding Asian patients were found to have delayed completion of primary series and booster vaccination compared with White patients. Black patients had the lowest and most delayed primary series (AHR, 0.76; 95% CI, 0.75-0.76), first-generation booster (AHR, 0.57; 95% CI, 0.56-0.57), and bivalent booster (AHR, 0.58; 95% CI, 0.57-0.60) uptake compared with White patients. Patients with at least 1 immunocompromising condition had higher (AHR, 1.62; 95% CI, 1.60-1.64) primary series coverage and earlier first-generation booster (AHR, 1.13; 95% CI, 1.12-1.15) and bivalent booster (AHR, 1.27; 95% CI, 1.25-1.29) uptake compared with those without immunocompromising conditions. There were also disparities in timing of primary series completion among patients with public compared with private health insurance (AHR, 0.37; 95% CI, 0.37-0.38) and patients younger than 30 years compared with patients aged 30 to 49 years.

**Table 2.** Adjusted Hazard Ratios for COVID-19 Vaccine Uptake Associated With Patient Demographic Characteristics

Characteristic	Adjusted hazard ratio (95% CI)		
	Primary series (n = 1 001 235)	First-generation booster (n = 628 891)	Bivalent booster (n = 635 539)
Primary language			
English	1 [Reference]	1 [Reference]	1 [Reference]
Non-English^a^	0.86 (0.85-0.87)	0.74 (0.72-0.75)	0.65 (0.64-0.67)
Country of origin			
US	1 [Reference]	1 [Reference]	1 [Reference]
Non-US^b^	1.21 (1.20-1.22)	0.99 (0.98-1.01)	0.99 (0.98-1.01)
Race and ethnicity			
Non-Hispanic American Indian or Alaska Native	0.92 (0.87-0.96)	0.81 (0.77-0.87)	0.90 (0.83-0.97)
Non-Hispanic Asian	1.26 (1.25-1.28)	1.17 (1.15-1.18)	1.03 (1.01-1.05)
Non-Hispanic Black or African American	0.76 (0.75-0.76)	0.57 (0.56-0.57)	0.58 (0.57-0.60)
Hispanic or Latino	0.95 (0.93-0.96)	0.85 (0.83-0.86)	0.83 (0.81-0.85)
Non-Hispanic Native Hawaiian or Pacific Islander	0.93 (0.85-1.01)	0.92 (0.83-1.02)	0.92 (0.80-1.05)
Non-Hispanic White	1 [Reference]	1 [Reference]	1 [Reference]
Multiracial	0.82 (0.81-0.82)	0.89 (0.88-0.89)	0.81 (0.80-0.82)
Unknown	0.73 (0.72-0.74)	0.84 (0.82-0.85)	0.79 (0.78-0.81)
Sex			
Female	1.13 (1.13-1.14)	1.04 (1.04-1.05)	1.09 (1.08-1.10)
Male	1 [Reference]	1 [Reference]	1 [Reference]
Unknown or other	0.90 (0.79-1.03)	1.29 (1.09-1.54)	1.77 (1.42-2.19)
Age, y^c^			
0.5-4	0.15 (0.15-0.16)	NA	0.92 (0.89-0.96)
5-11	0.38 (0.37-0.38)	0.28 (0.28-0.29)	0.93 (0.91-0.95)
12-16	0.61 (0.61-0.62)	0.59 (0.58-0.60)	0.89 (0.88-0.91)
17-29	0.79 (0.79-0.80)	0.73 (0.72-0.74)	0.66 (0.65-0.67)
30-49	1 [Reference]	1 [Reference]	1 [Reference]
50-64	1.25 (1.24-1.25)	1.29 (1.28-1.30)	1.38 (1.37-1.40)
≥65	2.17 (2.15-2.19)	1.99 (1.97-2.01)	2.40 (2.37-2.44)
Immunocompromising condition^d^			
No	1 [Reference]	1 [Reference]	1 [Reference]
Yes	1.62 (1.60-1.64)	1.13 (1.12-1.15)	1.27 (1.25-1.29)
Insurance			
Private	1 [Reference]	1 [Reference]	1 [Reference]
None or unknown	0.72 (0.71-0.72)	0.78 (0.77-0.79)	0.94 (0.93-0.95)
Public	0.37 (0.37-0.38)	0.56 (0.54-0.57)	0.41 (0.39-0.43)

Abbreviation: NA, not applicable (not included in model).

^a^ Includes those with unknown language (15 445 [1.5%]).

^b^ Includes those with unknown country of origin (266 157 [26.6%]).

^c^ Age calculated as of June 30, 2022.

^d^ Defined as at least 1 diagnosis of solid malignancy, hematologic malignancy, rheumatologic or inflammatory disorder, or organ or stem cell transplant; other intrinsic immune condition; or immunodeficiency between January 1, 2019, and June 30, 2023.^8^

Multivariable analysis evaluating disaggregated primary language found notable variation in vaccine uptake within specific linguistic groups (Table 3). Spanish, Somali, Vietnamese, and Hmong were the 4 most common non-English languages present in the data (1.5%, 1.5%, 0.5%, and 0.2%, respectively). Somali-speaking patients had a lower rate of primary series uptake (AHR, 0.74; 95% CI, 0.72-0.76) compared with English-speaking patients, with the disparities in uptake widening for first-generation (AHR, 0.34; 95% CI, 0.32-0.36]) and bivalent (AHR, 0.30; 95% CI, 0.27-0.32) booster uptake. Hmong-speaking patients had lower primary series uptake (AHR, 0.81; 95% CI, 0.78-0.86) compared with English-speaking patients. This was lower than the uptake for other Asian language categories. Primary series uptake was lowest for the language group that included languages from Central and Eastern Europe (AHR, 0.36; 95% CI, 0.34-0.39).

**Table 3.** AHRs for COVID-19 Vaccine Uptake Associated With Common Non-English Primary Languages

Primary language	Patients, No. (%)		AHR (95% CI)^a^		
	Total (N = 1 001 235)	Booster eligible (n = 635 539)	Primary series (N = 1 001 235)	First-generation booster (n = 628 891)	Bivalent booster (n = 635 539)
Hmong	2404 (0.2)	2829 (0.4)	0.81 (0.77-0.85)	0.81 (0.76-0.86)	0.72 (0.66-0.78)
Somali	15 407 (1.5)	5935 (0.9)	0.74 (0.72-0.76)	0.34 (0.32-0.36)	0.30 (0.27-0.32)
Spanish	15 459 (1.5)	8908 (1.4)	0.95 (0.93-0.97)	0.80 (0.77-0.83)	0.70 (0.66-0.73)
Vietnamese	5007 (0.5)	3903 (0.6)	0.92 (0.89-0.95)	0.95 (0.92-0.99)	0.67 (0.63-0.71)
Languages from Eastern Africa^b^	5397 (0.5)	2873 (0.5)	0.97 (0.94-1.01)	0.54 (0.51-0.58)	0.45 (0.41-0.50)
Languages from East Asia^c^	2548 (0.3)	1961 (0.3)	0.93 (0.89-0.97)	0.99 (0.93-1.04)	0.86 (0.80-0.92)
Languages from Southeast Asia^d^	4833 (0.5)	3541 (0.6)	0.9 (0.87-0.93)	0.82 (0.79-0.86)	0.87 (0.82-0.91)
Languages from Southern Asia and the Indian subcontinent^e^	1821 (0.2)	1285 (0.2)	0.88 (0.84-0.93)	0.93 (0.87-1.00)	0.85 (0.77-0.93)
Languages from Central and Eastern Europe^f^	1796 (0.2)	639 (0.1)	0.36 (0.34-0.39)	0.62 (0.56-0.69)	0.33 (0.28-0.39)
Languages from Northern Africa and the Middle East^g^	1714 (0.2)	990 (0.2)	0.91 (0.85-0.96)	0.69 (0.63-0.76)	0.78 (0.70-0.88)
Other	2466 (0.2)	1533 (0.2)	0.91 (0.87-0.96)	0.84 (0.79-0.90)	0.88 (0.81-0.96)
Unknown	15 445 (1.5)	7254 (1.1)	0.89 (0.87-0.91)	1 (0.96-1.03)	0.93 (0.89-0.97)

^a^ English was the reference category. Adjusted for patient sex, age, race and ethnicity, country of origin (US vs non-US), immunocompromised status, and insurance type.

^b^ Swahili, Amharic, Oromo, Tigrinya, Kinyarwanda, Luganda, Lingala, Kru, Sango, Dinka, Anuak, Nuer, Bassa, Kissii, Afar, Vai, Greybo, Greyboe, Madi, Mende, Rundi, and Kunama.

^c^ Mandarin Chinese, Cantonese, Korean, Japanese, Taiwanese, Taishanese, Mongolian, Uzbek, and Krahn.

^d^ Khmer (Cambodian), Burmese, Thai, Lao, Tagalog, Indonesian, Malaysian (Bahasa), Filipino, Tibetan, Visayan, Tulu, Ceblano, Central Khmer, Ilocano, Malay, Bahasa, Laotian, Cambodian/Khmer, and Karen.

^e^ Hindi, Nepali, Bengali, Urdu, Punjabi, Tamil, Telugu, Gujarati, Marathi, Sinhala, Malayalam, and Kannada.

^f^ Russian, Ukrainian, Polish, Romanian, Bulgarian, Serbian, Czech, Slovak, Hungarian, Estonian, Lithuanian, Latvian, Albanian, Georgian, Croatian, Abkhazian, Greek, Armenian, Bosnian, Azerbaijan, and Moldavian.

^g^ Arabic, Persian, Kurdish, Turkish, Azerbaijani, Pashto, Dari, and Hebrew.

In sensitivity analyses, when excluding the 268 503 patients with missing primary language or country of origin, estimates were similar to those from primary analyses for uptake of primary series completion (AHR, 0.84; 95% CI, 0.83-0.85), the first-generation booster (AHR, 0.75; 95% CI, 0.73-0.77), and the bivalent booster (AHR, 0.70; 95% CI, 0.68-0.72). Estimates for non–US-born patients were lower than in the primary analysis for uptake of the primary series (AHR, 1.18; 95% CI, 1.17-1.20]), the first-generation booster (AHR, 0.97; 95% CI 0.95-0.98), and bivalent booster (AHR, 1.00; 95% CI, 0.98-1.02). When including patients who did not complete a primary series in the bivalent analysis (n = 6846), primary series completion was associated with a higher likelihood of bivalent booster uptake (AHR, 21.7; 95% CI, 21.2-22.2).

## Discussion

In this cohort study of 1 001 235 patients receiving primary care, patients with a primary language other than English had both lower coverage and delays in receiving COVID-19 vaccines compared with those with English as their primary language. While primary series completion was higher among those with a country of origin outside the US compared with those from the US, coverage decreased for booster doses, with similar time to vaccination in both groups. Documenting the overall uptake of COVID-19 primary series and booster vaccines by race and ethnicity is helpful but only captures part of the story. Delays in vaccination result in longer time spent at risk for COVID-19 infection and its potential complications.^14^ This study highlights the importance of understanding vaccine uptake by language group in the US, as individuals with a non-English primary language were at greater risk for COVID-19.

Previous studies identified language and limited English proficiency as key demographic factors for evaluating and identifying health disparities.^5,15,16,17^ Culturally tailored approaches to public health messaging, especially reaching patients in their primary or preferred language, are increasingly being recognized as a critical component of health equity.^6,18^ Our study built on information in a previous study by Quadri et al^5^ by expanding the age range of patients to include the pediatric population and extending the study period to include booster vaccinations. We identified delayed times to completion of the primary series, first-generation booster, and bivalent booster for individuals with a primary language other than English, accounting for age, race and ethnicity, immunocompromising conditions, and insurance status. Additionally, we observed delays in vaccination for patients with publicly funded insurance, signaling a disparity aligning with socioeconomic factors. With use of the most recent National Immunization Survey Adult COVID Module 2021-2022, Ohlsen et al^19^ reported that Spanish-language interviewees had lower vaccination coverage than English-language interviewees and were significantly more likely than English-language interviewees to live below the federal poverty level, lack health insurance, and live in a county with a high Social Vulnerability Index. Non–English-language interviewees with primary language other than Spanish were also more likely than interviewees with English as their primary language to live below the federal poverty level and lack health insurance. Other possible explanations for delayed vaccination for those with a language preference other than English include lower access to health care services or information in their primary language, lower vaccine-seeking behavior, differences in outreach efforts to these communities for primary series vs booster vaccines, or differences in the rate of natural infection.^20^ These findings underscore some social issues that may impact health care access and point to specific needs for populations with limited English proficiency. Barriers to COVID-19 vaccination that have been identified from focus groups conducted in US Hispanic communities include, for example, language and literacy difficulties, cost concerns, immigration status and location or transportation limitations.^21,22^ As a result, workplace vaccinations and school involvement with vaccinations were noted facilitators of vaccination.^21^ Recent additional qualitative evidence among Spanish-speaking patients supports the suggestion that more convenient vaccination sites, such as local churches or pharmacies, may serve to increase self-efficacy of obtaining vaccinations more quickly.^22^

A key finding of this work was from the comparison between primary series and booster completion. Primary series vaccination coverage was lower for the non–English-speaking group compared with the English-speaking group but higher for those born outside the US compared with US-born patients. The reasons for this are unclear but may be related to the large population of non–US-born persons employed as health care workers as well as federal COVID-19 vaccination immigration requirements. Over 18% of US health care workers were born outside the US, with approximately 29% of physicians and 15% of nurses born outside the US.^23^ These individuals likely have English listed as their primary language, were some of the first groups targeted for COVID-19 vaccination, and were among the groups for whom COVID-19 vaccine mandates existed. Additionally, as of October 2021, federal immigration requirements included proof of primary series vaccination as part of the application for entry into the US if a World Health Organization–approved COVID-19 vaccine was available in a patient’s country of departure. However, we were unable to capture information about health care employment or date of US arrival and, thus, were not able to determine to what extent these 2 issues may be related to our findings.^24^

Delays in booster uptake by race and ethnicity have been identified in other COVID-19 vaccine booster uptake studies.^25^ As updated COVID-19 vaccines will likely be available annually, providing the entire population with up-to-date information about COVID-19 vaccine recommendations will be critical. Ensuring availability of linguistically appropriate information about vaccine recommendations and safety may help reduce delays in vaccination and promote health equity for patients with a language preference other than English.

### Limitations

Limitations of this study include the constraints of EHR data with limited race and ethnicity options, inconsistent documentation of country of origin and primary language, and lack of information on occupational factors that may have defined vaccine eligibility, all of which could bias our estimates of vaccination uptake. In addition, this cohort was limited to patients with at least 1 health care visit during the study period to collect more granular demographic data, such as primary language and need for an interpreter. However, this may have led to selection of patients who were already more health seeking and may limit the generalizability of our findings. Finally, some booster doses may have been missed due to the inclusion criteria for individuals in the booster cohort to have a completed primary series by December 31, 2022. While some individuals may have completed their primary series after December 31, 2022, and received a booster dose prior to the end of the study, this was likely limited.

## Conclusions

In this cohort study, patients with a non-English primary language had both lower coverage and delays in receiving COVID-19 vaccines compared with those with English as their primary language. These findings suggest that disaggregation of primary language is crucial for understanding disparities that exist by primary language and creating effective interventions to address these disparities. To attenuate delays in COVID-19 vaccination and reduce disparities in vaccine coverage, public health practitioners should consider how to include language-specific materials when designing or implementing interventions.

## Notes

Supplement.
Data Sharing Statement
