# Supplementary material for: Time to COVID-19 Vaccination by Language and Country of Origin
Source: JAMA Netw Open. 2024 Oct 3;7(10):e2437388. doi: 10.1001/jamanetworkopen.2024.37388 (PMC11450518; doi:10.1001/jamanetworkopen.2024.37388)
Supplement: Supplement. — Data Sharing Statement [file jamanetwopen-e2437388-s001.pdf]

Data Sharing Statement

Nolan. Time to COVID-19 Vaccination by Language and Country of Origin. *JAMA Netw Open*. Published October 03, 2024. doi:10.1001/jamanetworkopen.2024.37388

Data
Data available: Yes
Data types: Data dictionary, Other (please specify)
Additional Information: Summary data from participating sites
How to access data: margaret.b.nolan@healthpartners.com
When available: With publication

Supporting Documents
Document types: None

Additional Information
Who can access the data: Researchers whose proposed use of the data has been approved
Types of analyses: Specified purpose
Mechanisms of data availability: Signed data access agreement
